# Methodological Advances in Brain Connectivity

**DOI:** 10.1155/2012/492902

**Published:** 2012-09-06

**Authors:** Luca Faes, Ralph G. Andrzejak, Mingzhou Ding, Dimitris Kugiumtzis

**Affiliations:** ^1^Department of Physics and BIOtech, University of Trento, 38123 Mattarello, Italy; ^2^Department of Information and Communication Technologies, Universitat Pompeu Fabra, 08018 Barcelona, Spain; ^3^J. Crayton Pruitt Family Department of Biomedical Engineering, University of Florida, Gainesville, FL 32611-6131, USA; ^4^Department of Mathematical, Physical and Computational Sciences, Faculty of Engineering, Aristotle University of Thessaloniki, 54124 Thessaloniki, Greece

Determining how distinct neurons or brain regions are connected and communicate with each other is a crucial point in neuroscience, as it allows to investigate how the functional integration of specialized neural populations enables the emergence of coherent cognitive and behavioral states. The general concept of brain connectivity encompasses different aspects: structural connectivity is related to the description of anatomical pathways and synaptic connections; functional connectivity investigates statistical dependencies between spatially separated brain regions; effective connectivity refers to models aimed at elucidating driver-response relationships. The study of these different modes of brain connectivity is fundamental both for the investigation of the neurophysiological processes engaged in cognitive and perceptive processing and for the assessment of a variety of neurological disorders.

Over the last past few decades, the continuous advancement of multichannel data acquisition technologies has made it possible to collect neuroscience data at multiple levels of description, ranging from neural spikes to local field potentials and electroencephalography (EEG)-magnetoencephalography (MEG). This increasing data availability and the contemporaneous improvement of signal processing capabilities have contributed in placing increasing demands on methods for the quantitative assessment of neural interactions. Accordingly, a variety of methodological approaches have emerged for the estimation of brain connectivity from multivariate neurophysiological time series. These approaches build on different analysis frameworks which point out specific aspects of connectivity: linear parametric models are explicitly related to the frequency domain representation of multivariate data, thus favoring the assessment of connectivity for specific brain rhythms; information theory provides tools that describe both linear and nonlinear interactions and are free from the shortcomings of model specification; phase synchronization analysis characterizes the relation between the phases of different brain units seen as coupled oscillators, allowing detection of synchronization regardless of the relation between signal amplitudes. All these frameworks provide measures able to reflect the various aspects of brain connectivity, such as undirected measures of association (e.g., coherence, mutual information, phase synchronization index) and directed measures of Granger causality (e.g., partial directed coherence, transfer entropy).

To get new and deeper insights into the complex dynamics of interacting brain regions, the existing brain connectivity measures need to be refined, extended, and complemented with other tools. Common issues to be addressed are estimation problems arising in the presence of noise contamination and nonstationarity, significance assessment, distinguishing direct from indirect causal effects, and aspects related to the difficulty of performing full multivariate analyses over short data sets. An important problem of EEG/MEG recordings is that the connectivity patterns estimated at the sensor level may strongly differ from those really existing between the underlying neural sources, as a consequence of a mixing effect known as volume conduction. While a common approach to deal with this issue is to estimate connectivity after application of an inverse method for source localization, methodological advances are needed to improve the localization accuracy of ill-posed inverse problems. Moreover, once brain connectivity measures are computed, the formal representation of connectivity patterns in graph or matrix format leads to employ concepts of network analysis to interpret these patterns. Accordingly, the study of topological properties like clustering degree, modularity, and presence of network motifs is becoming increasingly popular for the investigation of the organizational principles of brain processes.

The papers of this special issue reflect the variety in the approaches for the estimation of brain connectivity described above, as well as the need to improve and adapt these approaches to the more and more demanding qualitative requirements and challenges of modern neurophysiological applications. J. Sun et al. offer a review of phase synchronization analysis methods for the inference of functional brain connectivity, describing definitions, estimation, and significance assessment, presenting some extensions, and discussing the issues that affect the detection of phase synchronization from neural data. C. Alvarado-Rojas and M. Le Van Quyen exploit phase synchronization analysis and clustering techniques to identify, from intracranial EEG recordings acquired in epileptic patients during seizure-free periods, dynamic modes of brain synchrony which are characteristic for the wake-sleep cycle. L. Faes et al. introduce a common framework for the unified description of the most popular frequency domain connectivity measures based on linear parametric modeling of multiple time series, discussing their relations, theoretical interpretation, advantages and limitations, and practical estimation. A. Brovelli assesses the reliability of frequency domain Granger causality analysis performed on a single-trial basis, showing that, when combined with parametric statistical tests, Granger causality spectra successfully recover causal interactions in both synthetic and neurophysiological data. Y. Liu and S. Aviyente illustrate the advantages over linear parametric Granger causality of an information theoretic tool, the directed information, as regards the assessment of directional connectivity in both simulated time series and EEG data. D. Marinazzo et al. face the important problem of estimating causality in complex brain networks through fully multivariate approaches; working in the frame of information theory, they provide a novel approach for partial conditioning to a subset of informative variables, showing that this approach can help to overcome computational and numerical problems otherwise arising with traditional full conditioning schemes.

P. Belardinelli et al. investigate in a realistic MEG environment the known localization bias due to correlation between source time series occurring for the popular beamformer inverse method, showing that this bias is relevant only for extremely high degrees of source correlation. F. Avarvand et al. combine subspace and beamformer source localization methods with estimation of the imaginary part of the coherence in simulated and real EEG data, implementing an approach that is sensitive to connectivity rather than to activity and, as such, improves localization accuracy and detection of source interactions. C. Micheli and C. Braun deal with the problem of characterizing connectivity between neural source activity and muscular activity after the localization of multiple correlated sources; when applied to MEG and electromyographic signals during a pinch grip task, their approach highlights patterns of corticomuscular coherence otherwise not obtainable with the use of standard methods. C. Schmidt et al. propose a new analytical approach to the detection of topological motifs in EEG connectivity networks estimated in patients with major depression during painful stimulation, suggesting that specific motifs may help explaining the relationship between pain and depression. A. Alvarellos-Gonzàlez et al. study structural brain connectivity at the synaptic level, presenting enhanced computational models of neural networks which evidence the potentiation of synaptic connectivity that takes place in the brain when glial cells are considered in addition to artificial neurons.

*Luca Faes* *Ralph G. Andrzejak* *Mingzhou Ding* *Dimitris Kugiumtzis*

